# Efficacy and challenges of anti-PD1 in MSI-H mCRC: a case report on concurrent infections and ir-AIHA

**DOI:** 10.3389/fonc.2024.1407312

**Published:** 2024-08-13

**Authors:** Xiaxia Pei, Jun Zhao, Ruiying Luo, Lijun Da, Enxi Li, Hao Zhu, Yanhong Li, Yaoting Luo, Kun Tian, Zhiping Wang, Feixue Song

**Affiliations:** ^1^ Department of Medical Oncology, Second Hospital of Lanzhou University, Lanzhou, China; ^2^ Department of General Surgery, Second Hospital of Lanzhou University, Lanzhou, China; ^3^ Department of Radiology, Second Hospital of Lanzhou University, Lanzhou, China; ^4^ Institute of Hematology, Second Hospital of Lanzhou University, Lanzhou, China; ^5^ Institute of Urology, Second Hospital of Lanzhou University, Key Laboratory of Urological Diseases in Gansu Province, Lanzhou, China

**Keywords:** high microsatellite instability, metastatic colorectal cancer, immunotherapy, bacteremia, liver fluke, immunotherapy-related autoimmune hemolytic anemia

## Abstract

Anti-programmed cell death protein 1 (PD-1) therapy has demonstrated notable efficacy in treating patients with deficient mismatch repair/high microsatellite instability (dMMR/MSI-H) metastatic colorectal cancer (mCRC). However, its clinical application is fraught with challenges and can lead to significant immune-related adverse events (ir-AEs). In this report, we present a complicated case of an mCRC patient with MSI-H and mutations in β2M and LRP1B proteins, complicated by concurrent bacteremia and liver fluke infection, who received first-line anti-PD1 therapy. The patient exhibited a positive response to anti-PD1 treatment, even in the presence of concomitant antibiotic and anti-parasitic interventions. Additionally, the patient experienced immunotherapy-related autoimmune hemolytic anemia (ir-AIHA), a rare hematological ir-AE, which was effectively treated later on. Immunotherapy represents a pivotal and highly effective approach to tumor treatment. Baseline assessment of the MMR and MSI status is a crucial step before initiating immunotherapy, and regular ongoing assessments during the treatment course can facilitate early recognition of any secondary complications, enabling prompt intervention and ensuring optimal therapeutic outcomes. Overall, a multidisciplinary diagnostic and therapeutic algorithm can help maximize the therapeutic benefits of immunotherapy.

## 1 Introduction

Colorectal carcinoma (CRC) is the third leading cancer in the world, with high mortality and morbidity rates (1), since about one-quarter of CRC cases are usually diagnostically confirmed in their advanced stages (2). For metastatic CRC (mCRC) patients, improving the overall survival (OS) and quality of life (QoL) are the primary therapeutic objectives. Currently, chemotherapy, targeted drugs and immuntherapy are the mainstream treatment options for these patients (3). Recent studies have unveiled that mCRC patients with pathologic hallmarks of deficient mismatch repair or high microsatellite instability are most likely to respond positively to immunotherapies (4). Pembrolizumab, a potential inhibitor of programmed death-1 (PD-1), has been approved by the US Food and Drug Administration (FDA) for this subtype of mCRC patients (5). Nevertheless, clinical conditions of mCRC patients tends to be intricate, often complicated by various factors such as intestinal obstruction, systemic infections, and malnutrition. Inevitably, these complexities significantly impact the treatment efficacy and outcomes, especially in immunotherapy. In the case report, we present the clinical findings of an mCRC patient with MSI-H and mutations in Beta 2-microglubulin (β2M) and lipoprotein receptor-related protein 1B (LRP1B), who was concomitantly treated for bacteremia and liver fluke infection. This patient exhibited a positive response to anti-PD1 therapy. Additionally this patient encountered a rare hematological adverse event called immunotherapy-related autoimmune hemolytic anemia (ir-AIHA), which was effectively treated later on.

## 2 Case presentation

The patient is 51 years old, middle-aged female, professional senior management, no prior history of chronic diseases and no family history of tumor. She underwent a laparoscopic radical resection of right colon on July 3^rd^, 2020. The postoperative pathological findings revealed aggressive ulcerative moderately differentiated adenocarcinoma in the colon. The pathological stage was Stage IIC (T4bN0M0) based on the American Joint Committee on Cancer, 8th Edition. Immunohistochemistry (IHC) results were as follows: MLH1(+), PMS-2(+), MSH-2(+), MSH-6(+), PD-L1(-), suggesting mismatch repair proficient (pMMR). Next-generation sequencing (NGS) analysis detected MSI-H, tumor mutation load (TMB) of 17.06 mutations/Mb, and wild-type status for BRAF/NRAS/KRAS, with mutations in β2M and LRP1B. Postoperatively, she received six cycles of the XELOX regimen (capecitabine plus oxaliplatin) from August to December 2020. I.

**Figure 1:**
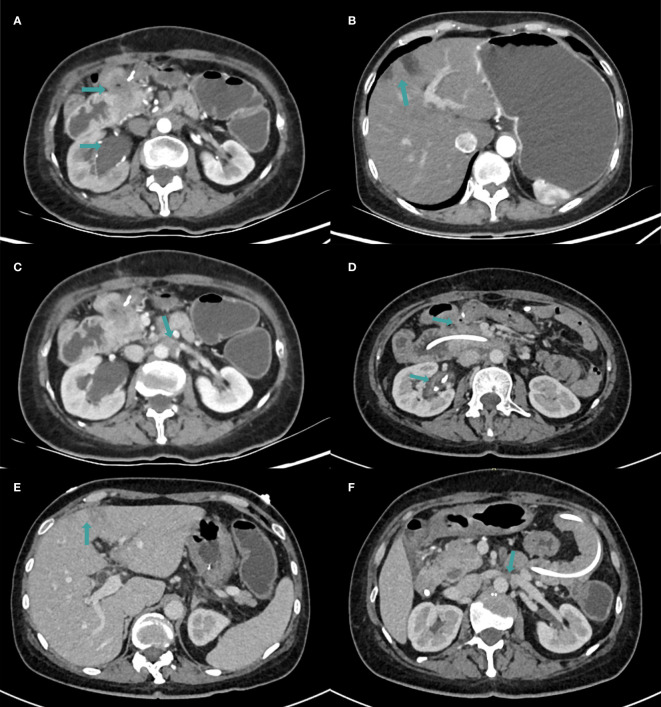
The CT examination findings. **(A–C)** CT images in January 2022. **(A)** The metastasis invaded the duodenum and right ureter, resulting in right hydronephrosis. **(B)** Multiple low-density lesions in the liver which were considered to be metastatic in nature. **(C)** Metastasis in the retroperitoneal lymph node. **(D–F)** CT images in April 2022 after 4 cycles of immunotherapy. **(D)** Duodenal lesions were significantly reduced and hydronephrosis was relieved. **(E)** The number of liver lesions was reduced. **(F)** Metastasis in the retroperitoneal lymph node was reduced.

**Figure 2:**
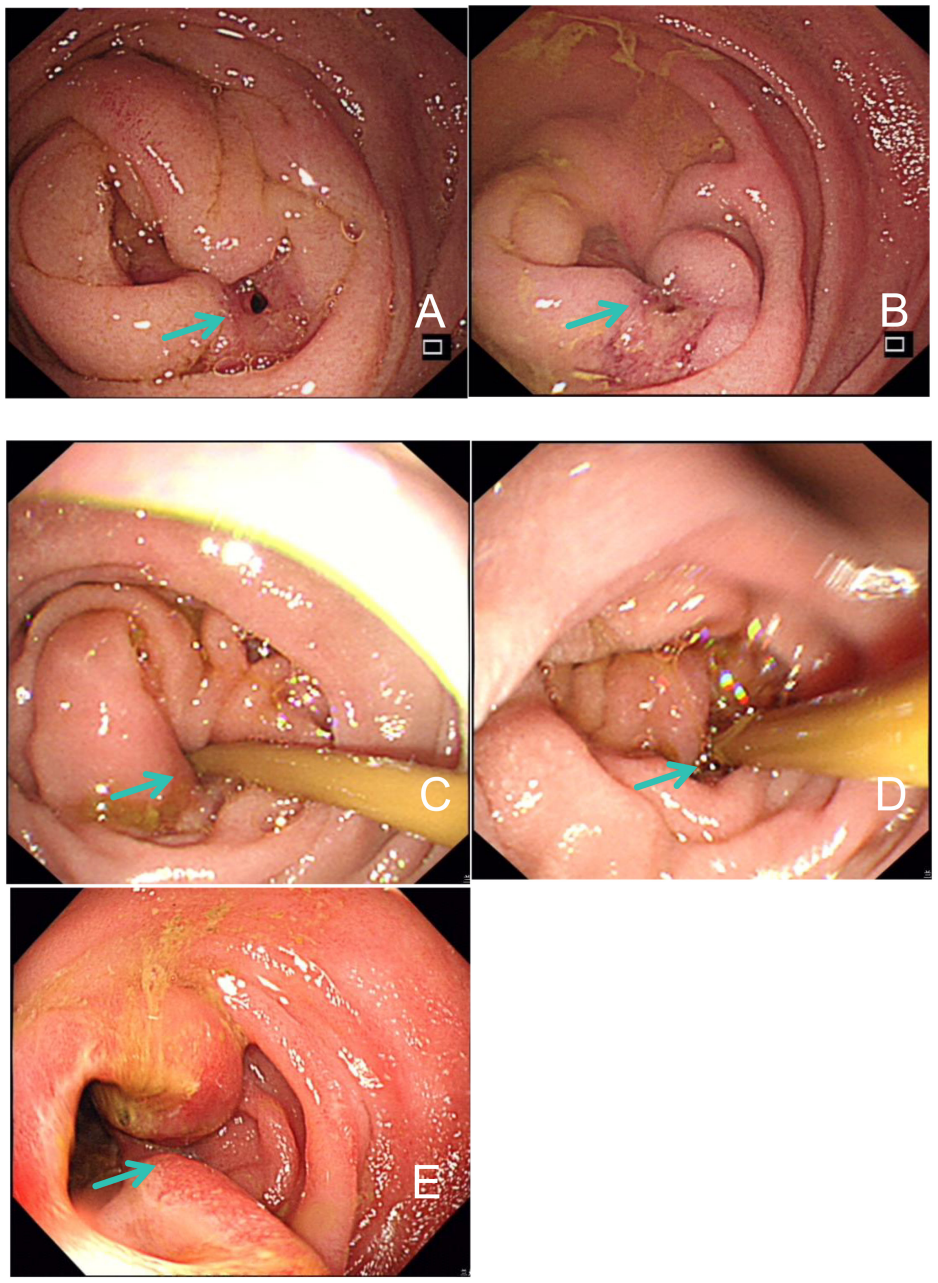
Gastroduodenoscopy examination findings. **(A, B)** Proliferative lesions and intestinal stenosis of duodenum on January 24, 2022. **(C, D)** Stenosis of the duodenal descending part was improved after 4 cycles of immunotherapy in April 2022. **(E)** Edema and ulcerative lesions in intestinal mucosa on March 2, 2023.

In December 2021, she experienced tumor recurrence and metastasis, leading to duodenal obstruction, right hydronephrosis, and severe malnutrition. (**Figures 1A–C**, **2A, B**). After the multidisciplinary treatment team(MDT) discussion, the patient was successfully inserted a nasojejunal nutrition tube by interventional radiology doctors to improve the nutritional status on January 26, 2022. Subsequently, enteral nutrition combined with parenteral nutrition support therapy was provided to the patient. Due to right ureteral metastasis-associated hydronephrosis, a right ureteral stent implantation procedure was performed by urologists on January 27, 2022. Following symptomatic treatments, the patient’s overall health condition was improved, with relief from vomiting and nausea symptoms.

Given her high MSI and TMB and inability to tolerate systemic chemotherapy, the pembrolizumab treatment was initiated on February 9, 2022. Afterward, she complained of sudden chills, high febrile temperature, and significantly increased levels of C-reactive protein (CRP), procalcin (PCT), and interleukin-6 (IL-6). In February 2022, the patient developed bacteremia and blood culture confirmed *Enterococcus faecalis* infection. Cefoperazone and sulbactam were administered for anti-infective treatment and probiotics were supplemented to reduce the impact of antibiotics on ICIs.

Subsequently, the patient was found to have clonorchis sinensis infection in a stool examination, and was treated with oral praziquantel. From April 2022, the patient continued to receive immunotherapy of pembrolizumab. After four cycles, a follow-up CT scan showed a reduction in duodenal lesions, retroperitoneal metastatic lymph nodes, and liver lesions (**Figures 1D–F**). Electronic gastroduodenoscopy revealed improvement in the duodenal descending segment stenosis (**Figures 2C, D**) and the naso-jejunal feeding tube was removed.

**Figure 3:**
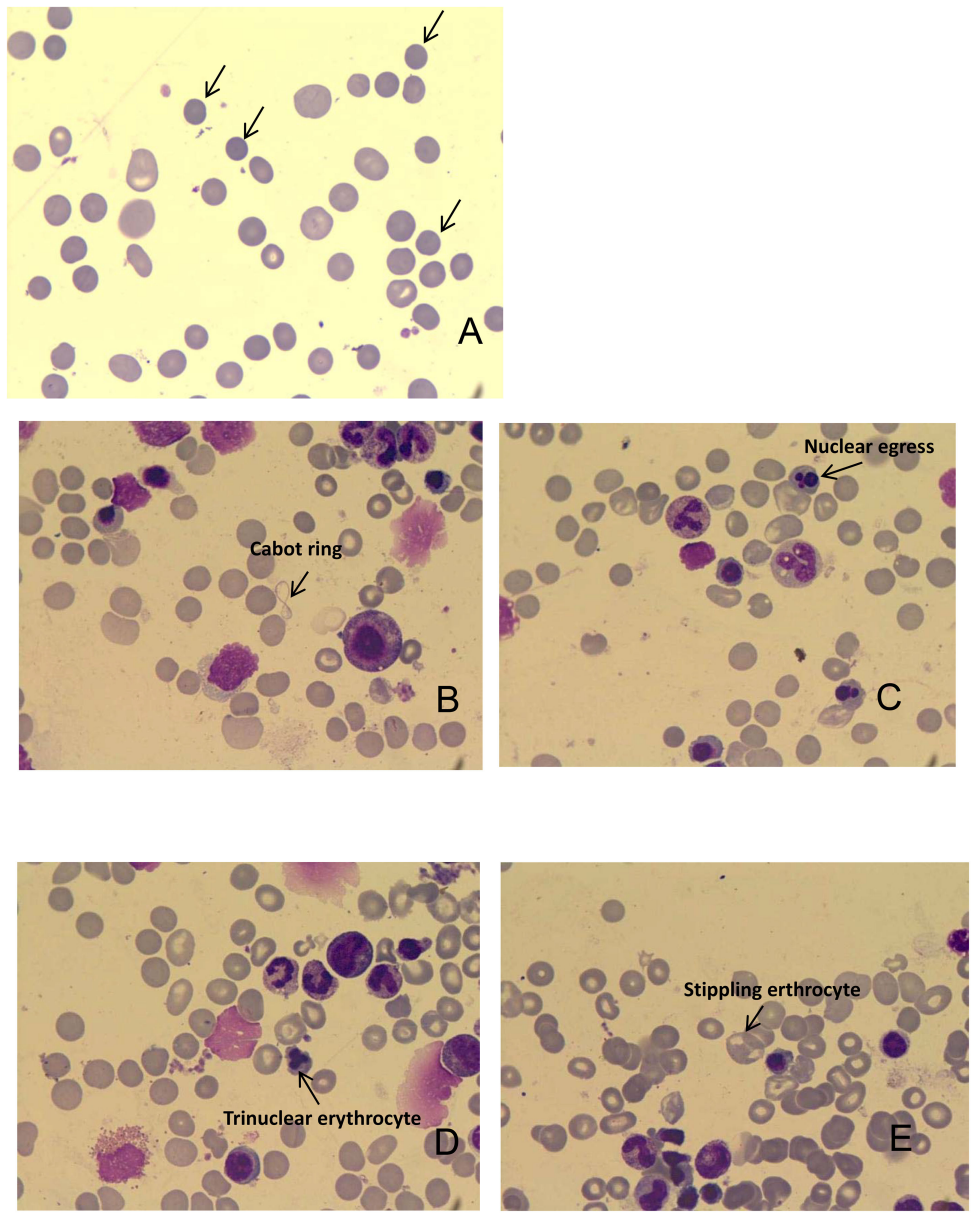
**(A)** Representative image shows the presence of spherical cells on peripheral blood smears. **(B)** Cabot ring in the bone marrow; **(C)** Nuclear egress in the bone marrow; **(D)** Trinuclear erythrocyte in the bone marrow; **(E)** Stippling erythrocyte in the bone marrow.

In February 2023, the patient’s hemoglobin (Hb) level dropped to 43 g/L, accompanied by dizziness and fatigue. Routine blood analysis revealed a significant increase in reticulocyte count (206.7 x 10^9/L) and reticulocyte ratio (0.10310). Additionally, the serum lactate dehydrogenase (LDH) level significantly increased to 486 U/L. Bone marrow (BM) aspiration analysis confirmed hemolytic anemia and the presence of spherocytes on peripheral blood smears (**Figures 3A–E**). Gastroscopy identified a stricture in the descending duodenum. Colonoscopy results showed mucosal edema and ulceration of the small intestine (**Figure 2E**), with biopsy revealing adenocarcinoma. IHC displayed MLH1 (+), PMS-2 (-), MSH-2 (+), and MSH-6 (+). The patient was diagnosed with ir AIHA, a rare hematological immune-related adverse event. Consequently, anti-PD1 therapy was ceased, and the patient received red blood cell (RBC) transfusions and intravenous methylprednisolone at 2 mg/kg per day. Post-treatment, her Hb level rebounded to 100 g/L. However, due to the severe toxicity of pembrolizumab, the patient discontinued anti-PD1 immunotherapy and underwent FOLFOX and TAS102 chemotherapy successively. Nonetheless, due to severe gastrointestinal adverse reactions, the patient discontinued these treatments in June 2023 and was treated with best supportive care from June to October 2023.

## 3 Discussion

The mCRC with dMMR/MSI-H is a highly immunogenic tumor subtype with unique pathological features such as right-sided primary, mucinous, poorly differentiated tumors and higher incidences of BRAF mutations (6). MSI-H accounts for about 15% of all CRCs and about 5% of stage IV cases (7). In patients with stage IV CRC, MSI status is a useful screening indicator for identifying suitable cancer patients for immune checkpoint inhibitor (ICI) therapy. ICIs have shown significant efficacy in MSI-H/dMMR CRC patients (8). Keynote 177 study showed that anti-PD1 antibody was superior to chemotherapy in terms of response rate and pathology-free survival (PFS) outcomes in first-line treatment of MSI-H CRC. Following that, pembrolizumab was recently approved by the FDA as a first-line treatment for mCRC management (9). Nivolumab plus ipilimumab also provided robust and durable clinical benefit in patients with MSI-H/dMMR mCRC (10). But the potential benefits of ICIs are limited to patients with MSI-H/dMMR, drug resistance and adverse events have hampered the therapeutic application of ICIs (11). MSI status not only be predicted by IHC analysis of MMR protein but also by polymerase chain reaction (PCR) or NGS. There may be inconsistent results between MMR-IHC and MSI by NGS. In the event of discordant results, pathologists should interpret any evidence of MMR deficiency by IHC or MSI by NGS or PCR as a positive result for patients to be eligible for immune checkpoint inhibitor therapy (12). Presently, only a small number of mCRC patients benefit from immunotherapy, so predictive markers are urgently needed to screen and expand the beneficiaries. Key molecular markers such as MMR, MSI, TMB, and levels of PD-L1 and DNA polymerase epsilon (POLE)/DNA polymerase delta 1 (POLD1) have demonstrated good predictive effects on the efficacy of immunotherapy for mCRC patients. In the NGS report of this patient, mutations in β2M and LRP1B had also been identified. β2M is the main component of major histocompatibility complex I (MHC-I), which is involved in antigen presentation and T-cell activation (13). It’s been shown that β2M mutation can lead to poor treatment outcomes in cancer immunotherapy, due to inhibition of antigen presentation (14). The latest findings suggest that TMB can be significantly increased in mCRC patients with MSI-H/dMMR tumors due to β2M mutation, which in turn might improve response to anti-PD1 therapy (15, 16). Patients with LRP1B mutations exhibit higher TMB scores (17) and are associated with improved outcomes of cancer immunotherapy (18). LRP1B mutation can facilitate immune cell infiltration and immune gene expressions in the tumor microenvironment (TME) (19). So, an accurate determination of MSI and MMR status is critical before embarking on immunotherapy. In this case, while IHC indicated pMMR, NGS analysis revealed MSI-H, TMB-H, and mutations in β2M and LRP1B, suggesting her potential benefit from anti-PD1 therapy, which indeed elicited a positive response.

During immunotherapy, the patient’s advanced CRC, low immunity, intestinal obstruction, and imbalance of intestinal flora led to damage to the intestinal epithelial barrier function, resulting in translocation of intestinal flora and bacteremia. The patient inevitably received antibiotic treatment. Some recent studies have shown that antibiotics can reduce the survival and response to ICIs in patients with advanced-stage solid tumors (20–22). Additionally, a recent study has demonstrated a strong association between antibiotics and worsened OS and PFS in cancer patients undergoing ICIs immunotherapy, particularly within the 60 days preceding the initiation of ICI and the following 30 days (23). However, several studies have also reported that oral Bifidobacterium supplementation can enhance dendritic cell maturation, increase tumor-specific CD8+ T-cell responses in the TME, and restore the antitumor efficacy of PD-L1 blocking (24). In addition to bifidobacterium, enterococcus and faecalibacterium may also play a role as an immune adjuvant in ICI immunotherapy (25, 26). As a result, this patient received probiotic treatment which not only restored balance to the intestinal flora but also mitigated the potential impacts of antibiotics on ICIs. Recently, clinical trials are focusing on the critical role of the microbiome in cancer research. Emerging evidence suggests that the diversity of gut microbiome can influence anti-tumor immunity, and predict treatment efficacy and toxicity level of ICI immunotherapy (27, 28). The diversity and composition of the gut microbiota are positively associated with anti-PD-1 treatment responses in melanoma patients, as well (29). The study of Mirji demonstrates that microbial metabolites can enable tumor-associated macrophage (TAM) to become immunogenic and promote effector T-cell activity, transforming the TME to an immune-activated state (30). Tanoue et al. isolated a consortium of 11 bacterial strains from fecal samples of healthy human donors that could strongly induce interferon-γ(IFN-γ)-producing CD8+ T-cells in the gut and enhance the therapeutic efficacy of ICIs in genotypic tumor models (31). The findings of Xu have also confirmed that changes in the gut microbiome can lead to the modulation of glycerophospholipid metabolism, which may affect the expression of immune-related cytokines like IFN-γ and IL-2 in the TME, resulting in a different therapeutic effect of anti-PD-1 antibody (32). Therefore, gut microbiota-based tumor immunotherapy can be a promising and effective approach to cancer therapy in the future.

In addition to the primary condition, the patient also suffered from a parasitic liver fluke infection. Due to a compromised immune state, most cancer patients are at an elevated risk of parasitic and microbial infections (33). Parasitic infections not only cause infectious diseases but also may induce tumorigenesis (34). The WHO’s International Agency for Research on Cancer has classified liver fluke as a Group I carcinogen for cholangiocarcinoma (35). The typical imaging findings of liver flukes are multiple, ill-defined, fleeting hypodense or hypoechoic areas in the liver (36). Presently, the detection of liver fluke eggs in fecal samples using microscopic examination, PCR-based assays, and fluke antigen detection is still the primary diagnosis for liver fluke infections (37). Clinical treatment mainly relies on drug therapy, with common medications including albendazole, praziquantel and so on. Albendazole can also inhibit tumor growth *in vitro* and *in vivo* (38). It can inhibit tubulin polymerization and the production of vascular endothelial growth factor (VEGF), promoting apoptosis of tumor cells via oxidative stress and DNA breakage (39, 40). To date, albendazole has been repositioned as a promising anticancer drug. However, studies have revealed that parasitic infections may have antagonistic effects on tumor growth by overcome cancer immunosuppressive microenvironment (41). The effects of parasites on tumors may include inducing apoptosis, activating immune responses, avoiding metastasis and angiogenesis, inhibiting proliferation signals, and regulating inflammatory responses that promote cancer (42). Therefore, parasites or their antigens have the potential to be used as anti-cancer therapeutics (43). Following the anti-parasitic treatment, we found that CD4+ and CD4+/CD8+ T-cells were significantly increased in the peripheral blood, while the CD4+/CD25+ regulatory T-cell population was significantly decreased. We speculated that dead fluke-secreted antigens might stimulate the proliferation and differentiation of CD4+ T-cells, simultaneously reducing the regulatory T-cell infiltration, which modulated the TME and was conducive to tumor control possibly.

Although the patient responded well to the immunotherapy, she also experienced severe ir-AE. The frequency of hematological ir-AEs caused by ICIs is 3.6% for all grades and 0.7% for Grades III-IV, but the mortality rate is close to 14% (44). Usually, the hematological irAEs mainly appeared as immune thrombocytopenia, pancytopenia or aplastic anemia, neutropenia, AIHA, bicytopenia, pure red cell aplasia (PRCA), and cytokine release syndrome (45). The occurrence time of hem-irAEs is around 0.9–198.0 weeks and the median time to onset is about 10.1 weeks (46). The ir-AIHA symptoms are defined according to the criteria defined by Leaf et al.:1) Sudden decrease in hemoglobin by greater than 2g/dL; 2) at least two Laboratory features of hemolysis (elevated serum lactate dehydrogenase; increased reticulocyte percentage or absolute count; low or undetectable serum haptoglobin; the presence of spherical cells on peripheral blood smears); 3) AIHA occurs after ICIs initiation; 4) exclusion of other causes of anemia; 5)ICIs treatment is considered to be the most likely cause of AIHA (47). A bone marrow aspiration examination can help rule out the possibility of tumor cell infiltration into the bone marrow. ICIs therapy should be discontinued for Grades 3–4 toxicities with the initiation of high-dose corticosteroids (prednisone 1–2mg/kg/d) and RBC transfusion. The addition of immunosuppressive drugs such as rituximab and intravenous immunoglobulin can be taken into account if no improvement is observed in initial treatments (48, 49). As the risk of recurrence of irAEs after ICIs rechallenge, the rechallenge should be carefully considered. The patient was diagnosed ir-AIHA according to the diagnostic criteria and her symptoms were improved after treatment of prednisone and RBC transfusions. Although the incidence of Hem-irAEs is relatively low, the associated mortality rate can be significantly high. With increasing clinical applications of ICIs, heightened awareness of this potentially severe and life-threatening autoimmune complication is essential. Prompt detection and appropriate management can lead to favorable outcomes in such cases.

**Figure 4:**
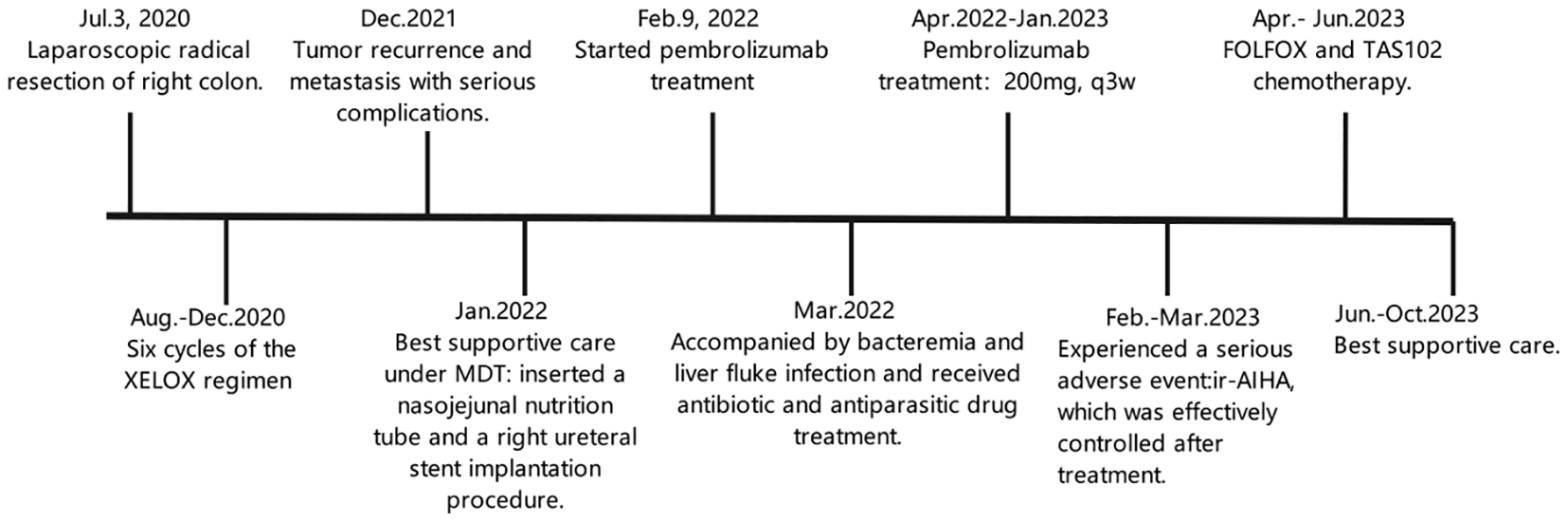
Timeline.

Before treatment, we confirmed that the patient was MSI-H through comprehensive testing, who was a beneficiary of immunotherapy, and the tumor was effectively controlled during the initial phase of treatment. Throughout the treatment process of this patient, an MDT comprising of experts in medical oncology, surgical oncology, gastroenterology, interventional surgery, urology, nutrition, infection, hematology, infectious diseases, pharmacy, pathology, imaging, and clinical laboratory collaborated to formulate an effective and personalized treatment plan to improve the patient’s QoL. Unfortunately, due to serious adverse events, we did not try immunotherapy again, and the patient could not tolerate chemotherapy in the later stage, resulting in tumor progression (**Figure 4**). So in the process of tumor treatment, we need to further identify and establish predictive indicators for the efficacy and adverse events of ICIs.

## 4 Conclusion

Tumor immunotherapy stands as a pivotal and effective approach to cancer treatments. Nonetheless, its clinical application has been challenged by uncertain efficacy, intricate complications, and potentially severe adverse reactions. To address these complexities, several key considerations need to be implemented urgently. First, the determination of MSI and MMR status is critical before embarking on immunotherapy. In cases where IHC indicates proficient MMR, it is strongly advisable to employ NGS for a comprehensive assessment of MSI and TMB status. This aids in identifying the most suitable candidates for immunotherapy. Second, conducting a thorough baseline evaluation of patients before commencing immunotherapy is imperative. Furthermore, the implementation of regular monitoring for various related therapies throughout the treatment course is essential for early detection and timely intervention in cases of abnormal or unprecedented outcomes. Finally, the MDT facilitates a collaborative and holistic approach to patient care and can be tailored to individual patient-specific needs. MDT plays a pivotal role in the management of CRC and provides more precise, efficient, and safe care to achieve maximum benefits for patients grappling with advanced stages of the disease.

## Data Availability

The original contributions presented in the study are included in the article/supplementary material. Further inquiries can be directed to the corresponding authors.

## References

[1] Sung H Ferlay J Siegel RL Laversanne M Soerjomataram I Jemal A . Global cancer statistics 2020: GLOBOCAN estimates of incidence and mortality worldwide for 36 cancers in 185 countries. CA Cancer J Clin. (2021) 71:209–49. doi: 10.3322/caac.21660 33538338

[2] Sevčíková K Ušáková V Bartošová Z Sabol M Ondrusova M Ondrus D . Chirurgická liečba metastáz a jej vplyv na prognózu u pacientov s metastatickým kolorektálnym karcinómom [Surgical treatment of metastases and its impact on prognosis in patients with metastatic colorectal carcinoma]. Klin Onkol. (2014) 27:38–44. doi: 10.14735/amko201438 24635436

[3] Mukherji R Weinberg BA Pedersen KS . Immunotherapy for colorectal cancer. Hematol Oncol Clin North Am. (2022) 36:603–26. doi: 10.1016/j.hoc.2022.02.010 35577706

[4] Choucair K Radford M Bansal A Park R Saeed A . Advances in immune therapies for the treatment of microsatellite instability-high/deficient mismatch repair metastatic colorectal cancer (Review). Int J Oncol. (2021) 59:74. doi: 10.3892/ijo.2021.5254 34396449 PMC8360619

[5] André T Shiu KK Kim TW Jensen BV Jensen LH Punt C . Pembrolizumab in microsatellite-instability-high advanced colorectal cancer. N Engl J Med. (2020) 383:2207–18. doi: 10.1056/NEJMoa2017699 33264544

[6] Ward R Meagher A Tomlinson I O’Connor T Norrie M Wu R . Microsatellite instability and the clinicopathological features of sporadic colorectal cancer. Gut. (2001) 48:821. doi: 10.1136/gut.48.6.821 11358903 PMC1728324

[7] Mulet-Margalef N Linares J Badia-Ramentol J Jimeno M Sanz-Monte C Manzano-Mozo J . Challenges and therapeutic opportunities in the dMMR/MSI-H colorectal cancer landscape. Cancers (Basel). (2023) 15:1022. doi: 10.3390/cancers15041022 36831367 PMC9954007

[8] Alouani E Mercier M Flecchia C Auclin E Hollebecque A Mazard T . Efficacy of immunotherapy in mismatch repair-deficient advanced colorectal cancer in routine clinical practice. AGEO study ESMO Open. (2023) 8:101574. doi: 10.1016/j.esmoop.2023.101574 37244250 PMC10265605

[9] Casak SJ Marcus L Fashoyin-Aje L Mushti SL Cheng J Shen YL . FDA approval summary: pembrolizumab for the first-line treatment of patients with MSI-H/dMMR advanced unresectable or metastatic colorectal carcinoma. Clin Cancer Res. (2021) 27:4680–4. doi: 10.1158/1078-0432.CCR-21-0557 PMC8416693 33846198

[10] André T Lonardi S Wong KYM Lenz HJ Gelsomino F Aglietta M . Nivolumab plus low-dose ipilimumab in previously treated patients with microsatellite instability-high/mismatch repair-deficient metastatic colorectal cancer: 4-year follow-up from CheckMate 142. Ann Oncol. (2022) 33:1052–60. doi: 10.1016/j.annonc.2022.06.008 35764271

[11] Yan S Wang W Feng Z Xue J Liang W Wu X . Immune checkpoint inhibitors in colorectal cancer: limitation and challenges.Ann Oncol. Front Immunol. (2024) 15:1403533. doi: 10.3389/fimmu.2024.1403533 38919624 PMC11196401

[12] Vikas P Messersmith H Compton C Sholl L Broaddus R Davis A . Mismatch repair and microsatellite instability testing for immune checkpoint inhibitor therapy. ASCO endorsement of College of American Pathologists guideline. J Clin Oncol. (2023) 41:1943–8. doi: 10.1200/JCO.22.02462 36603179

[13] Yeon Yeon S Jung SH Jo YS Sol Jo Y Choi EJ Kim MS . Immune checkpoint blockade resistance-related B2M hotspot mutations in microsatellite-unstable colorectal carcinoma. Pathol Res Pract. (2019) 215:209–14. doi: 10.1016/j.prp.2018.11.014 30503610

[14] Wang H Liu B Wei J . Beta2-microglobulin(B2M) in cancer immunotherapies: Biological function, resistance and remedy. Cancer Lett. (2021) 517:96–104. doi: 10.1016/j.canlet.2021.06.008 34129878

[15] Liu F Zhong F Wu H Che K Shi J Wu N . Prevalence and associations of beta2-microglobulin mutations in MSI-H/dMMR cancers. Oncologist. (2023) 28:e136–44. doi: 10.1093/oncolo/oyac268 PMC10020813 36724040

[16] Zhang C Li D Xiao B Zhou C Jiang W Tang J . B2M and JAK1/2-mutated MSI-H colorectal carcinomas can benefit from anti-PD-1 therapy. J Immunother. (2022) 45:187–93. doi: 10.1097/CJI.0000000000000417 PMC8986629 35343934

[17] Yu G Mu H Fang F Zhou H Li H Wu Q . LRP1B mutation associates with increased tumor mutation burden and inferior prognosis in liver hepatocellular carcinoma. Med (Baltimore). (2022) 101:e29763. doi: 10.1097/MD.0000000000029763 PMC9239668 35777027

[18] Brown LC Tucker MD Sedhom R Sedhom R Schwartz EB Zhu J . LRP1B mutations are associated with favorable outcomes to immune checkpoint inhibitors across multiple cancer types. J Immunother Cancer. (2021) 9:e001792. doi: 10.1136/jitc-2020-001792 33653800 PMC7929846

[19] He Z Feng W Wang Y Shi L Gong Y Shi Y . LRP1B mutation is associated with tumor immune microenvironment and progression-free survival in lung adenocarcinoma treated with immune checkpoint inhibitors. Transl Lung Cancer Res. (2023) 12:510–29. doi: 10.21037/tlcr-23-39 PMC10088005 37057124

[20] von Itzstein MS Gonugunta AS Sheffield T Sheffield T Homsi J Dowell JE . Association between antibiotic exposure and systemic immune parameters in cancer patients receiving checkpoint inhibitor therapy. Cancers (Basel). (2022) 14:1327. doi: 10.3390/cancers14051327 35267634 PMC8909108

[21] Crespin A Le Bescop C de Gunzburg J Vitry F Zalcman G Cervesi J . A systematic review and meta-analysis evaluating the impact of antibiotic use on the clinical outcomes of cancer patients treated with immune checkpoint inhibitors. Front Oncol. (2023) 13:1075593. doi: 10.3389/fonc.2023.1075593 36937417 PMC10019357

[22] Tinsley N Zhou C Tan G Rack S Lorigan P Blackhall F . Cumulative antibiotic use significantly decreases efficacy of checkpoint inhibitors in patients with advanced cancer. Oncologist. (2020) 25:55–63. doi: 10.1634/theoncologist.2019-0160 31292268 PMC6964118

[23] Zhou J Huang G Wong WC Hu DH Zhu JW Li R . The impact of antibiotic use on clinical features and survival outcomes of cancer patients treated with immune checkpoint inhibitors. Front Immunol. (2022) 13:968729. doi: 10.3389/fimmu.2022.968729 35967438 PMC9367677

[24] Sivan A Corrales L Hubert N Williams JB Aquino-Michaels K Earley ZM . Commensal Bifidobacterium promotes antitumor immunity and facilitates anti-PD-L1 efficacy. Science. (2015) 350:1084–9. doi: 10.1126/science.aac4255 PMC4873287 26541606

[25] Griffin ME Espinosa J Becker JL Luo JD Carroll TS Jha JK . Enterococcus peptidoglycan remodeling promotes checkpoint inhibitor cancer immunotherapy. Science. (2021) 373:1040–6. doi: 10.1126/science.abc9113 PMC9503018 34446607

[26] Chaput N Lepage P Coutzac C Soularue E Le Roux K Monot C . Baseline gut microbiota predicts clinical response and colitis in metastatic melanoma patients treated with ipilimumab. Ann Oncol. (2017) 28:1368–79. doi: 10.1093/annonc/mdx108 28368458

[27] Elkrief A Derosa L Zitvogel L Kroemer G Routy B . The intimate relationship between gut microbiota and cancer immunotherapy. Gut Microbes. (2019) 10:424–8. doi: 10.1080/19490976.2018.1527167 PMC6546322 30339501

[28] Andrews MC Duong CPM Gopalakrishnan V Iebba V Chen WS Derosa L . Gut microbiota signatures are associated with toxicity to combined CTLA-4 and PD-1 blockade. Nat Med. (2021) 27:1432–41. doi: 10.1038/s41591-021-01406-6 PMC11107795 34239137

[29] Matson V Fessler J Bao R Chongsuwat T Zha Y Alegre M . The commensal microbiome is associated with anti-PD-1 efficacy in metastatic melanoma patients. Science. (2018) 359:104–8. doi: 10.1126/science.aao3290 PMC6707353 29302014

[30] Mirji G Worth A Bhat SA Sayed ME Kannan T Goldman AR . The microbiome-derived metabolite TMAO drives immune activation and boosts responses to immune checkpoint blockade in pancreatic cancer. Sci Immunol. (2022) 7:eabn0704. doi: 10.1126/sciimmunol.abn0704 36083892 PMC9925043

[31] Tanoue T Morita S Plichta DR Skelly AN Suda W Sugiura Y . A defined commensal consortium elicits CD8 T cells and anti-cancer immunity. Nature. (2019) 565:600–5. doi: 10.1038/s41586-019-0878-z 30675064

[32] Xu X Lv J Guo F Li J Jia Y Jiang D . Gut microbiome influences the efficacy of PD-1 antibody immunotherapy on MSS-type colorectal cancer via metabolic pathway. Front Microbiol. (2020) 11:814. doi: 10.3389/fmicb.2020.00814 32425919 PMC7212380

[33] Jeske S Bianchi TF Moura MQ Baccega B Pinto NB Berne ME . Intestinal parasites in cancer patients in the South of Brazil. Braz J Biol. (2018) 78:574–8. doi: 10.1590/1519-6984.175364 29185612

[34] Mandong BM Ngbea JA Raymond V . Role of parasites in cancer. Niger J Med. (2013) 22:89–92. 23829116

[35] Sripa B Deenonpoe R Brindley PJ . Co-infections with liver fluke and Helicobacter species: A paradigm change in pathogenesis of opisthorchiasis and cholangiocarcinoma? Parasitol Int. (2017) 66:383–9. doi: 10.1016/j.parint.2016.11.016 PMC5457716 27919744

[36] Dietrich CF Kabaalioglu A Brunetti E Richter J . Fasciolosis. Z Gastroenterol. (2015) 53:285–90. doi: 10.1055/s-0034-1385728 25860578

[37] Prueksapanich P Piyachaturawat P Aumpansub P Ridtitid W Chaiteerakij R Rerknimitr R . Liver fluke-associated biliary tract cancer. Gut Liver. (2018) 12:236–45. doi: 10.5009/gnl17102 PMC5945254 28783896

[38] Chai JY Jung BK Hong SJ . Albendazole and mebendazole as anti-parasitic and anti-cancer agents: an update. Korean J Parasitol. (2021) 59:189–225. doi: 10.3347/kjp.2021.59.3.189 34218593 PMC8255490

[39] Chen H Weng Z Xu C . Albendazole suppresses cell proliferation and migration and induces apoptosis in human pancreatic cancer cells. Anticancer Drugs. (2020) 31:431–9. doi: 10.1097/CAD.0000000000000914 32044795

[40] Petersen JSSM Baird SK . Treatment of breast and colon cancer cell lines with anti-helmintic benzimidazoles mebendazole or albendazole results in selective apoptotic cell death. J Cancer Res Clin Oncol. (2021) 147:2945–53. doi: 10.1007/s00432-021-03698-0 PMC11802096 34148157

[41] Adah D Yang Y Liu Q Gadidasu K Tao Z Yu S . Plasmodium infection inhibits the expansion and activation of MDSCs and Tregs in the tumor microenvironment in a murine Lewis lung cancer model. Cell Commun Signal. (2019) 17:32. doi: 10.1186/s12964-019-0342-6 30979375 PMC6461823

[42] Callejas BE Martínez-Saucedo D Terrazas LI . Parasites as negative regulators of cancer. Biosci Rep. (2018) 38:BSR20180935. doi: 10.1042/BSR20180935 30266743 PMC6200699

[43] Yousefi M Akbari M Hadipour M Dehkordi AB Farahbakhsh Z Darani HY . Parasites as potential targets for cancer immunotherapy. J Cancer Res Clin Oncol. (2023) 149:8027–38. doi: 10.1007/s00432-023-04694-2 PMC11797331 36949175

[44] Kramer R Zaremba A Moreira A Ugurel S Johnson DB Hassel JC . Hematological immune related adverse events after treatment with immune checkpoint inhibitors. Eur J Cancer. (2021) 147:170–81. doi: 10.1016/j.ejca.2021.01.013 33706206

[45] Michot JM Lazarovici J Tieu A Champiat S Voisin AL Ebbo M . Haematological immune-related adverse events with immune checkpoint inhibitors, how to manage? Eur J Cancer. (2019) 122:72–90. doi: 10.1016/j.ejca.2019.07.014 31634647

[46] Delanoy N Michot JM Comont T Kramkimel N Lazarovici J Dupont R . Haematological immune-related adverse events induced by anti-PD-1 or anti-PD-L1 immunotherapy: a descriptive observational study. Lancet Haematol. (2019) 6:e48–57. doi: 10.1016/S2352-3026(18)30175-3 30528137

[47] Leaf RK Ferreri C Rangachari D Mier J Witteles W Ansstas G . Clinical and laboratory features of autoimmune hemolytic anemia associated with immune checkpoint inhibitors. Am J Hematol. (2019) 94:563–74. doi: 10.1002/ajh.25448 PMC9552038 30790338

[48] Brahmer JR Lacchetti C Schneider BJ Atkins MB Brassil KJ Caterino JM . Management of immune-related adverse events in patients treated with immune checkpoint inhibitor therapy: American society of clinical oncology clinical practice guideline. J Clin Oncol. (2018) 36:1714–68. doi: 10.1200/JCO.2017.77.6385 PMC6481621 29442540

[49] Wilson NR Lockhart JR Garcia-Perdomo HA Oo TH Rojas-Hernandez CM . Management and outcomes of hematological immune-related adverse events: systematic review and meta-analysis. J Immunother. (2022) 45:13–24. doi: 10.1097/CJI.0000000000000390 34469413

